# Body composition and environmental factors’ influence on metabolically unhealthy obesity in children: insights from the CCACH study

**DOI:** 10.3389/fnut.2025.1620166

**Published:** 2025-10-02

**Authors:** Xiangjun Xie, Hong Cheng, Jingfan Xiong, Yan Li, Pei Xiao, Hongbo Dong, Xinying Shan, Yanyan Li, Liwan Fu, Jie Mi

**Affiliations:** ^1^Center for Non-Communicable Disease Management, Beijing Children’s Hospital, National Center for Children’s Health, Capital Medical University, Beijing, China; ^2^Department of Epidemiology, Capital Institute of Pediatrics, Beijing, China; ^3^Child and Adolescent Chronic Disease Prevention and Control Department, Shenzhen Center for Chronic Disease Control, Shenzhen, China; ^4^Key Laboratory of Major Diseases in Children, Ministry of Education, Beijing, China

**Keywords:** metabolic syndrome, metabolically healthy obesity, metabolically unhealthy obesity, body composition, life behavior factor

## Abstract

**Background:**

Metabolically unhealthy obesity (MUO) is a subtype of obesity characterized by alterations in metabolic health parameters, such as elevated glycemia, dyslipidemia, and increased blood pressure. Body composition, lifestyle behaviors, and their interactions influence its development. This study examined the impact of body composition and lifestyle factors on MUO phenotypes in children and adolescents with obesity.

**Methods:**

The study included 1,375 children with obesity, aged 6–19 years, from China. All participants underwent dual-energy X-ray absorptiometry (DXA) for the evaluation of body composition. Participants were classified as having MUO based on either insulin resistance (IR) or cardiometabolic risk (CR) factors (blood pressure, lipids, and glucose). The study also assessed the influence of body composition in conjunction with environmental factors, including lifestyle aspects and family medical history.

**Results:**

The prevalence of MUO-CR and MUO-IR was 43.6 and 38.9%, respectively. Android fat mass (FM), visceral fat, and fat-free mass (FFM) were identified as significant independent predictors of MUO, regardless of the CR or IR definitions used. For MUO-CR, a significant interaction was found between fast food consumption, FM, and FFM. For MUO-IR, sleep duration exhibited a notable interaction with FFM and FM across different regions. No comparable effect was observed for physical activity.

**Conclusion:**

This study suggests that the prevalence of MUO is below 50% in Chinese children with obesity. The results reveal that body composition and lifestyle factors are significantly linked to MUO status.

## 1 Introduction

Surveys indicate that the obesity rate among children and adolescents has surged approximately fourfold from 1990 to 2022, affecting a total of 159 million individuals (1). Obesity is a chronic, multifactorial disease characterized by excessive adiposity and is associated with comorbidities such as type 2 diabetes, dyslipidemia, and hypertension. It is increasingly regarded as a heterogeneous disease with diverse prognoses and outcomes. The metabolic phenotype concept was first proposed by Jean Vague (2) in 1950. Within this context, obesity can be classified into two distinct forms: metabolically healthy obesity (MHO) and metabolically unhealthy obesity (MUO). Individuals with MHO have obesity but exhibit no substantial metabolic abnormalities. They have a lower risk of developing conditions such as cardiovascular disease and diabetes than those with MUO, who are at an elevated risk for these same diseases (3). Importantly, MHO and MUO differ considerably in terms of disease progression, prognosis, and response to treatment, with MUO often requiring more aggressive metabolic management than MHO. The MHO phenotype is considered transient. Consequently, differentiating between these two forms of obesity is crucial for identifying individuals with MUO, who may require more intensive interventions than those with MHO. Moreover, recognizing the factors that contribute to the development of metabolic dysfunction in obesity is vital for effective prevention and treatment strategies (4).

Currently, there is no universally recognized criterion for defining MHO and MUO in children (5). Nonetheless, the majority of the studies rely on two primary criteria to distinguish between these conditions: the presence of cardiometabolic risk (CR) factors or insulin resistance (IR) (6). Individuals with MHO tend to exhibit better adipose tissue function and a more favorable fat distribution than those with MUO (7). Furthermore, using body mass index (BMI) remains a practical and widely used screening tool; however, it has proven inadequate as a sole metric for evaluating individual health and disease risk (8), as it fails to account for variations in body fat and muscle mass (9). Consequently, research on body composition, particularly in children and adolescents in the context of MHO, often focuses on the roles of visceral adipose tissue (VAT) and subcutaneous adipose tissue (SAT). This emphasis has sparked ongoing debates and controversies in the field. For instance, Zhan et al. (10) reported that MHO had a higher SAT but a lower VAT than MUO. Similarly, a recent study indicated that, within the same age group, individuals with MHO had lower levels of both VAT and SAT than those with MUO (11). To more precisely assess the impact of fat and muscle distribution across various body regions on the MHO phenotype, our study employed dual-energy X-ray absorptiometry (DXA) for precise measurement. Additionally, we expanded our analysis to include other areas of fat and muscle distribution, aiming to provide a more comprehensive understanding of the factors contributing to MHO and MUO.

On the other hand, recent studies on children and adolescents have highlighted the role of dietary behaviors in the pathogenesis of MHO. However, research on the effects of physical activity and sleep on MHO remains limited. Some studies have identified interactions between genetics and lifestyle factors (12), as well as associations between fat mass and health-related quality of life (HRQoL) (13) in children with MHO phenotypes. Notably, the combined influence of body composition and lifestyle factors (diet, physical activity, and sleep) on MHO/MUO status and their potential interactions remain unexplored. Given that body composition or lifestyle factors may not fully explain the transition from MHO to MUO, it is essential to investigate how these lifestyle factors interact with body composition to influence metabolic outcomes.

Therefore, this study aims to (1) investigate the independent associations of specific body composition characteristics (assessed by DXA, including regional fat and muscle distribution) and lifestyle behaviors on MUO status and (2) examine whether lifestyle factors modify the association between body composition and MUO status. We hypothesize that healthier lifestyle behaviors (e.g., balanced diet, adequate physical activity, and sufficient sleep) attenuate the adverse metabolic effects of unfavorable body composition profiles (e.g., high visceral adiposity), thus lowering the risk of MUO.

## 2 Methods

### 2.1 Study design

The China Child and Adolescent Cardiovascular Health (CCACH) project is a comprehensive, cross-sectional population investigation carried out from 2013 to 2015 to evaluate the cardiovascular health of urban children and adolescents in China (14). We selected four northern cities (Beijing, Changchun, Jinan, and Yinchuan) and three southern cities (Shanghai, Chongqing, and Chengdu), all of which are municipalities directly under central government jurisdiction or provincial capitals. In each city, schools were randomly selected to ensure a representative sample in terms of age, sex, and socioeconomic status. In total, 11,435 children underwent dual-energy X-ray absorptiometry (DXA).

The Ethical Review Committee of the Capital Institute of Pediatrics approved this study (approval number: 2012062), and all participants or their guardians provided written informed consent prior to participation.

### 2.2 General information and laboratory measurements

Before the research began, we developed a unified operation manual for all the measurement procedures. All participants removed their shoes and wore single-layer trousers during the examination. All assessments were conducted by qualified health professionals following a standardized protocol, as described in our previous study (15, 16). Height and weight were measured using a calibrated digital scale (Jianmin II, China Institute of Sport Science, Beijing, China) with a precision of 0.1 kg and 0.1 cm. Waist circumference was measured to the nearest 0.1 cm. Height, weight, and waist circumference measurements were obtained consecutively and averaged. Body mass index (BMI) was calculated as weight (kg) divided by the square of height (m^2^).

Systolic blood pressure (SBP) and diastolic blood pressure (DBP) were measured by trained personnel using a calibrated automatic electronic blood pressure monitor (OMRON HEM-7012, Omron Corporation, Kyoto, Japan). Each child’s resting blood pressure was measured three times, and the mean of the last two measurements was recorded for analysis. If the difference between two consecutive readings exceeded 10 mm Hg, additional measurements were obtained.

After a 10-h overnight fast, blood samples were collected from the antecubital veins using ethylenediaminetetraacetic acid (EDTA)-anticoagulated vacuum tubes.

Samples were centrifuged, aliquoted, and stored at −80 °C until analysis. Total cholesterol (TC), triglycerides (TG), and insulin levels were quantified using enzyme-linked immunosorbent assay (ELISA, Quantikine, R&D Systems, MN, USA). High-density lipoprotein cholesterol (HDL-C) and low-density lipoprotein cholesterol (LDL-C) were measured via a direct method (Sekisui Medical, Tokyo, Japan). Fasting plasma glucose (FPG) was determined by a hexokinase assay (BIOSINO, Beijing, China). Biochemical parameters were analyzed using an automated analyzer (Sekisui Medical, Tokyo, Japan), with an interassay coefficient of variation of <6.0% for all measurements. The Homeostasis Model Assessment of Insulin Resistance (HOMA-IR) was calculated as follows: [Fasting insulin (mU/L) × FPG (mmol/L)]/22.5.

### 2.3 Body composition measurements

All participants were assessed using Hologic Discovery (A, W, and Wi) fan–beam densitometers. The APEX 4.0 software (Hologic, Bedford, MA, USA) was used to segment the body into anatomical regions, including the head and limbs. The android region, defined as the abdominal area, extends from the midpoint of the lumbar spine at the waist to the superior border of the pelvis. The gynoid region (hip area) is defined as the region between the femoral head and the midpoint of the thigh. Appendicular skeletal muscle mass (ASMM) was assessed and used as the primary indicator of muscle mass, calculated as the sum of lean mass from the arms and legs (ASMM = ASMM_arm + ASMM_leg). Whole-body and regional fat mass (FM, kg) and fat-free mass (FFM, kg) were quantified.

### 2.4 Assessment of environmental factors

The questionnaire was designed and validated through pilot testing. For children under 12 years of age, the questionnaire was completed with assistance provided by their parents or guardians, whereas children aged 12 years and older self-reported their responses independently. Data were collected on demographic characteristics (sex, date of birth, and ethnicity), lifestyle factors (dietary habits, physical activity, and sleep patterns), early life conditions (preterm birth and birth weight), family medical history (parental obesity, and hypertension), and socioeconomic status (parental education level and annual household income). All participants were asked about their menarche or spermarche experiences and categorized by pubertal stage based on self-reported Tanner criteria.

Table 1 presents dietary intake records and their evaluation based on daily consumption frequency. Fast food was defined as fried items, including churros, fried chicken, hamburgers, and similar products. To facilitate interpretation, food items were categorized into two groups based on median frequency. For analytical clarity, food groups were dichotomized according to median consumption frequency. Physical activity was assessed through self-reported frequency and duration of moderate-to-vigorous physical activity (MVPA) during the preceding 12 months. The WHO recommends ≥60 min of daily MVPA as adequate for children and adolescents; participants not meeting this threshold were classified as insufficient (17). Sedentary behavior was further categorized based on daily sitting duration (<2 h vs. ≥2 h).

**Table 1:** Comparison of basic characteristics of MHO and MUO under different definitions.

Characteristics	CR Definition			IR Definition		
	MHO (*n* = 766)	MUO (*n* = 591)	*p*-value	MHO (*n* = 546)	MUO (*n* = 347)	*p*-value
Boys, %	65.67	65.48	0.990	65.57	62.25	0.348
Puberty, %	33.94	34.86	0.769	28.02	42.07	<0.001
Age, years	10.55 ± 3.56	11.1 ± 3.68	0.005	10.42 ± 3.57	11.6 ± 3.75	<0.001
BMI z-score	−0.22 ± 0.86	0.21 ± 1.11	<0.001	−0.07 ± 0.98	0.17 ± 1.17	0.001
WC z-score	−0.01 ± 1.02	0.01 ± 0.98	0.768	0.13 ± 0.97	0 ± 1.07	0.074
FM z-score	−0.07 ± 1.00	0.1 ± 0.98	0.003	−0.07 ± 0.98	0.16 ± 1.06	0.001
ANDROID_FM z-score	−0.13 ± 0.98	0.18 ± 1.00	<0.001	−0.05 ± 0.99	0.14 ± 1.04	0.005
GYNOID_FM z-score	−0.01 ± 0.99	0.02 ± 1.01	0.592	−0.09 ± 0.95	0.13 ± 1.09	0.001
TRUNK_FM z-score	−0.12 ± 0.98	0.17 ± 1.01	<0.001	−0.07 ± 0.98	0.19 ± 1.05	<0.001
FM_limb z-score	−0.01 ± 1.02	0.02 ± 0.97	0.681	−0.06 ± 0.97	0.11 ± 1.06	0.013
FM_leg z-score	0.02 ± 1.02	−0.01 ± 0.96	0.563	−0.05 ± 0.97	0.09 ± 1.05	0.040
FM_arm z-score	−0.06 ± 0.99	0.09 ± 1.01	0.004	−0.09 ± 0.97	0.13 ± 1.13	0.002
VAT z-score	−0.07 ± 0.97	0.11 ± 1.03	0.001	−0.09 ± 0.98	0.17 ± 1.04	<0.001
SAT z-score	−0.07 ± 1.00	0.10 ± 0.99	0.001	−0.01 ± 1.03	0.06 ± 0.92	0.293
FFM z-score	0.06 ± 1.00	−0.09 ± 0.98	0.005	0.08 ± 0.98	−0.16 ± 1.06	0.001
ANDROID_FFM z-score	−0.04 ± 1.03	0.03 ± 0.95	0.205	−0.06 ± 0.99	−0.13 ± 1.02	0.248
GYNOID_FFM z-score	−0.03 ± 1.03	0.02 ± 0.95	0.393	−0.07 ± 1.00	−0.13 ± 1.00	0.425
TRUNK_FFM z-score	−0.03 ± 1.01	0.03 ± 0.97	0.262	−0.06 ± 0.99	−0.19 ± 1.00	0.070
ASMM z-score	0.01 ± 1.00	−0.03 ± 0.99	0.466	−0.02 ± 0.98	−0.2 ± 1.00	0.010
ASMM_leg z-score	0.03 ± 1.01	−0.04 ± 0.98	0.209	−0.01 ± 0.98	−0.17 ± 1.00	0.015
ASMM_arm z-score	−0.03 ± 0.98	0.02 ± 1.01	0.371	−0.05 ± 0.97	−0.21 ± 0.98	0.017
MVPA≥1 h, %	23.76	26.57	0.263	24.54	23.63	0.818
Sedentary time ≥2 h/day, %	33.39	37.13	0.237	27.29	38.19	0.003
Sleep duration z-score	−0.02 ± 0.98	0.02 ± 1.02	0.543	0.09 ± 0.97	−0.02 ± 0.99	0.130
sufficient sleeping, n(%)	67.49	63.46	0.177	70.58	65.71	0.174
somnipathy, n(%)	28.26	29.15	0.798	26.48	22.88	0.298
Beans ≥2 times/week, %	61.55	65.58	0.201	65.06	68.29	0.412
Meat ≥7 times/week, %	54.97	55.06	1.000	61.20	60.75	0.965
Seafood ≥1 time/week, %	61.29	59.08	0.502	66.07	62.15	0.316
Dairy ≥7 times/week, %	56.72	54.47	0.570	66.67	57.21	0.035
Vegetables ≥9 times/week, %	50.65	56.35	0.042	61.36	58.21	0.387
Fruit ≥7 times/week, %	59.18	60.00	0.836	68.49	68.06	0.966
Soft drinks ≥1 time/week, %	47.08	56.02	0.005	47.60	53.19	0.165
Snack ≥1 time/week, %	63.62	64.90	0.725	61.47	62.85	0.767
Fast food ≥1 time/week, %	50.00	59.58	0.009	53.80	62.07	0.080
SBP, mm Hg	111.04 ± 10.89	123.29 ± 13.16	<0.001	114.74 ± 13.29	117.3 ± 13.82	0.007
DBP, mm Hg	65.15 ± 6.73	72.53 ± 9.26	<0.001	67.54 ± 8.8	69.03 ± 8.85	0.016
TC, mmol/L	3.99 ± 0.72	4.15 ± 0.85	<0.001	4.16 ± 0.77	4.08 ± 0.76	0.117
TG, mmol/L	0.76(0.52,1.07)	1.10(0.73,1,61)	<0.001	0.92(0.58,1.25)	0.84(0.59,1.24)	0.549
LDL-C, mmol/L	2.36 ± 0.64	2.54 ± 0.7	<0.001	2.48 ± 0.65	2.47 ± 0.64	0.799
HDL-C, mmol/L	1.33 ± 0.24	1.21 ± 0.27	<0.001	1.30 ± 0.27	1.27 ± 0.25	0.103
FG, mmol/L	5.16 ± 0.7	5.56 ± 0.70	<0.001	5.28 ± 0.80	5.58 ± 0.73	<0.001
Insulin, mU/L	8.44(5.93,12.19)	8.45(6.00,12.26)	0.586	6.45(5.15,7.89)	13.18(11.22,18.66)	<0.001
HOMA-IR	1.89(1.31,2.74)	2.10(1.50,3.09)	0.004	1.54(1.19,1.88)	3.28(2.71,4.46)	<0.001
Father with obesity, %	19.50	22.06	0.352	21.20	21.33	1.000
Mother with obesity, %	7.33	13.47	0.002	8.99	11.93	0.249
Father with hypertension,%	13.83	15.33	0.590	13.60	15.22	0.670
Mother with hypertension, %	7.72	9.69	0.359	8.62	10.50	0.548
Father’s education university or higher, %	29.79	27.13	0.362	42.21	24.57	<0.001
Mother’s education university or higher, %	25.65	22.56	0.262	35.73	19.86	<0.001

MHO, metabolically healthy obesity; MUO, metabolically unhealthy obesity; CR, cardiometabolic risk; IR, insulin resistance; BMI, indicates body mass index; WC, waist circumference; FM, fat mass; FFM, fat-free mass; VAT, visceral adipose tissue; SAT, subcutaneous adipose tissue; ASMM, appendicular skeletal muscle mass; MVPA, moderate-to-vigorous physical activity; SBP, systolic blood pressure; DBP, diastolic blood pressure; TC, total cholesterol; TG, triglycerides; LDL-C, low-density lipoprotein cholesterol; HDL-C, high-density lipoprotein cholesterol; HOMA-IR, homeostasis model assessment of insulin resistance. For normally distributed variables, data are presented as ^−^x ± s, and p-values were calculated using the independent samples t-test. For skewed variables, data are expressed as median (interquartile range), and p-values were derived from the Mann–Whitney U test.

Total daily sleep duration includes both nocturnal and daytime sleep. According to the National Sleep Foundation, sleep duration below the following thresholds is classified as insufficient: fewer than 9 h per day for children aged 6–13 years, fewer than 8 h for adolescents aged 14–17 years, and fewer than 7 h for adults aged 18 years or older (18). Experiencing a delay of over 30 min in achieving sleep is categorized as having difficulty initiating sleep. Sleep disorders were assessed based on self-reported symptoms, including snoring, mouth breathing, breath cessation, somnambulism, nightmares, limb jerking, bedwetting, difficulty rising in the morning, or nocturnal headaches.

### 2.5 Definitions of metabolically healthy and unhealthy obesity

The obesity threshold was established in accordance with the Chinese National Standard WS/T 586–2018 (19). We employed two standard definitions (SDs) to classify children and adolescents with obesity into MHO and MUO phenotypes. The assessment of the following traditional CR should be conducted initially (12): (1) waist circumference (WC) ≥ 90th percentile for age and sex; (2) SBP and/or DBP ≥ 90th percentile for age and sex; (3) TG levels of ≥1.12 mmol/L for individuals under 10 years and ≥1.46 mmol/L for those over 10 years; (4) HDL-C levels ≤1.03 mmol/L; and (5) FPG levels ≥5.6 mmol/L. MHO-CR was defined as obesity accompanied by one or fewer cardiometabolic risk factors, whereas MUO-CR was defined as obesity with two or more cardiometabolic risk factors. The second definition depends on the existence or non-existence of IR, and there is no universally recognized threshold for IR in children and adolescents. Based on a study conducted in China (20), we determined that the most suitable cut-off value for our cohort was 2.3. Accordingly, MHO-IR was defined as HOMA-IR ≤ 2.3, and MUO-IR was defined as HOMA-IR > 2.3.

### 2.6 Statistical analysis

Continuous variables were tested for normality using histogram methods and the Shapiro–Wilk test. Those conforming to a normal distribution are presented as ^−^x ± s and compared using an independent sample *t*-test; non-normally distributed variables are expressed as median (interquartile range, IQR) and analyzed with the Mann–Whitney U test. Categorical variables are reported as proportions (%) and assessed with the chi-square tests. To account for age-related physiological variations (6–18 years), we standardized BMI, WC, and body composition measures using regression-based z-scores (internally studentized residuals). For each outcome variable (e.g., BMI), a linear regression model was fitted with age as the predictor:


$$Z_{i}=\frac{e_{i}}{\hat{\sigma }\sqrt{1-h_{\mathrm{ii}}}},$$

where,

e_i_ = raw residual (observed value—model-predicted value),

$\;\hat{\sigma }$ = estimated standard deviation of residuals, and.

h_ii_ = leverage value (a measure of how far an observation deviates from the mean predictor values).

Logistic regression was employed to examine the relationship between each variable and the MHO phenotype, controlling for age, sex, region, and pubertal development. A multi-factor test was subsequently conducted by incorporating all pertinent MHO factors into the final logistic regression model. In the multivariate regression analyses, variance inflation factors (VIFs) were used to evaluate multicollinearity among independent variables. VIF values ≥ 10 indicate severe multicollinearity, suggesting such variables should not be retained simultaneously in the model.

Lifestyle factors and body composition indicators are more likely to influence metabolic health in a multiplicative manner (that is, the effect of one factor changes proportionally with the level of the other factor) rather than a simple additive effect. Therefore, logistic regression analyses were employed to examine the multiplicative interaction between DXA measures and lifestyle factors by including a product term (e.g., FM × Sleep duration) in logistic regression models. Lifestyle factors were entered as bicategorical variables, with adjustments made for age, sex, region, and pubertal development. The analysis used R version 4.3.2 software. A two-tailed *p*-value of < 0.05 was considered statistically significant.

## 3 Results

### 3.1 Fundamental characteristic

**Figure 1:**
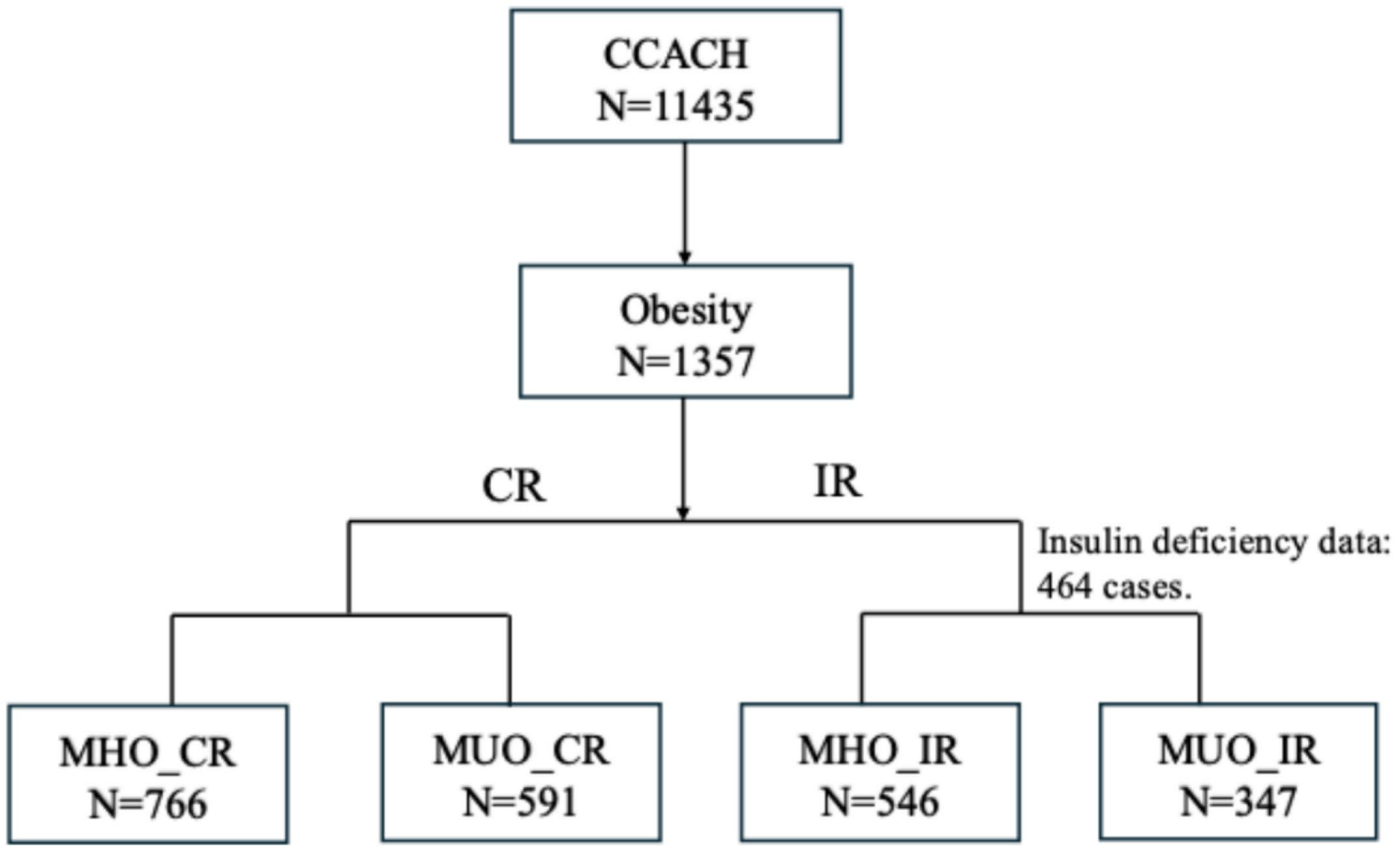
Sample selection flowchart of CCACH (2013–2015). Among the 1357 children with obesity, 893 were randomly selected to undergo measurement of insulin levels. CCACH, the China Child and Teenager Cardiovascular Health study; MHO, metabolically healthy obesity; MUO, metabolically unhealthy obesity; CR, cardiometabolic risk; IR, insulin resistance.

This study analysed 1,357 participants aged 6–19 years (65.6% male; mean age 10.8 ± 3.6 years). Figure 1 shows the screening flowchart. Table 1 presents demographic and clinical characteristics of participants stratified by MHO and MUO phenotypes according to both definitions. Based on the CR definition, 43.6% of children with obesity were classified as MUO, while 38.9% met MUO criteria by IR thresholds. Among the total cohort, 175 (19.6%) were MUO by both CR and IR definitions, whereas 309 (34.6%) were MHO across both definitions.

The MHO group demonstrated better cardiometabolic profiles. Compared to participants with MUO, the MHO group was younger and had lower BMI, FM, and VAT, and higher FFM. MHO defined by CR criteria was associated with healthier dietary patterns (lower soft drinks and fast food intake) and lower maternal obesity prevalence. MHO, defined by IR criteria, correlated with delayed pubertal onset, reduced sedentary behavior, higher skeletal muscle mass, increased dairy consumption, and higher parental education levels.

Table 2 presents the factors associated with MUO phenotypes after adjusting for age, sex, pubertal development, and residence. Body composition factors, namely FM, android FM, arm FM, VAT, and FFM, significantly predicted MUO under both definitions (all *p* < 0.03). In contrast, ASMM, arm ASMM, and leg ASMM showed no significant association with MUO status under either definition (all *p* > 0.05). Among the behavioral factors, soft drinks (*p* = 0.016), fast food (*p* = 0.026), and a mother with obesity (*p* = 0.002) emerged as the most significant determinants of MUO, as defined by CR. Sleep duration (*p* = 0.052) and parental education level (*p* < 0.010) were significant factors correlated with MUO, as defined by IR.

**Table 2:** Correlation between MUO and lifestyle, body composition, etc., as defined by CR and IR.

	CR			IR		
	OR	95% CI	*p*-value	OR	95% CI	*p*-value
BMI z-score	1.60	1.42–1.81	<0.001	1.26	1.10–1.44	0.001
WC z-score	1.01	0.90–1.13	0.861	0.92	0.80–1.06	0.246
FM z-score	1.22	1.09–1.37	0.001	1.20	1.04–1.38	0.015
ANDROID_FM z-score	1.43	1.27–1.61	<0.001	1.17	1.02–1.34	0.029
GYNOID_FM z-score	1.03	0.92–1.16	0.586	1.18	1.02–1.37	0.025
TRUNK_FM z-score	1.42	1.27–1.61	<0.001	1.23	1.07–1.42	0.005
FM_limb z-score	1.03	0.92–1.15	0.644	1.13	0.99–1.31	0.082
FM_leg z-score	0.96	0.86–1.08	0.496	1.11	0.96–1.28	0.160
FM_arm z-score	1.21	1.08–1.36	0.001	1.17	1.02–1.35	0.026
VAT z-score	1.28	1.14–1.44	<0.001	1.23	1.07–1.43	0.005
SAT z-score	1.30	1.14–1.48	<0.001	1.00	0.85–1.18	0.966
FFM z-score	0.83	0.74–0.93	0.001	0.84	0.73–0.97	0.018
ANDROID_FFM z-score	1.08	0.97–1.21	0.178	0.95	0.82–1.09	0.467
GYNOID_FFM z-score	1.05	0.94–1.17	0.408	1.00	0.87–1.15	0.973
TRUNK_FFM z-score	1.07	0.96–1.2	0.232	0.91	0.79–1.04	0.173
ASMM z-score	0.95	0.85–1.07	0.427	0.89	0.76–1.03	0.106
ASMM_leg z-score	0.93	0.83–1.04	0.177	0.90	0.78–1.04	0.140
ASMM_arm z-score	1.06	0.94–1.19	0.319	0.88	0.76–1.03	0.107
MVPA≥1 h, %	1.08	0.84–1.39	0.562	0.94	0.68–1.30	0.718
Sedentary time ≥2 h/day, %	1.07	0.82–1.41	0.607	1.31	0.92–1.87	0.132
Sleep duration z-score	1.06	0.94–1.19	0.360	0.86	0.74–1.00	0.052
sleep sufficiency, n(%)	0.96	0.73–1.27	0.775	1.04	0.73–1.48	0.823
somnipathy, n(%)	1.02	0.78–1.33	0.897	0.78	0.54–1.10	0.157
Beans ≥2 times/week, %	1.14	0.88–1.48	0.334	1.10	0.79–1.53	0.566
Meat ≥7 times/week, %	0.96	0.75–1.24	0.776	0.96	0.70–1.32	0.813
Seafood ≥1 time/week, %	0.93	0.72–1.19	0.544	0.81	0.58–1.11	0.192
Dairy ≥7 times/week, %	0.91	0.69–1.22	0.539	0.90	0.61–1.32	0.575
Vegetables ≥9 times/week, %	1.13	0.90–1.42	0.283	0.94	0.70–1.25	0.649
Fruit ≥7 times/week, %	1.02	0.79–1.32	0.859	1.12	0.81–1.57	0.490
Soft drinks ≥1 time/week, %	1.36	1.06–1.74	0.016	1.11	0.81–1.51	0.522
Snack ≥1 time/week, %	1.06	0.81–1.38	0.686	0.85	0.61–1.17	0.314
Fast food ≥1 time/week, %	1.39	1.04–1.87	0.026	1.31	0.90–1.92	0.163
Father with obesity, %	1.14	0.84–1.54	0.400	0.97	0.66–1.40	0.857
Mother with obesity, %	1.94	1.28–2.96	0.002	1.21	0.73–2.00	0.458
Father with hypertension,%	1.08	0.74–1.57	0.694	1.08	0.66–1.74	0.764
Mother with hypertension, %	1.22	0.75–1.96	0.423	1.09	0.60–1.95	0.780
Father’s education university or higher, %	0.85	0.64–1.13	0.275	0.60	0.42–0.84	0.004
Mother’s education university or higher, %	0.84	0.62–1.13	0.243	0.59	0.40–0.85	0.005

OR was adjusted for age, sex, puberty, and region. MUO, metabolically unhealthy obesity; CR, cardiometabolic risk; IR, insulin resistance; BMI, indicates body mass index; WC, waist circumference; FM, fat mass; FFM, fat-free mass; VAT, visceral adipose tissue; SAT, subcutaneous adipose tissue; ASMM, appendicular skeletal muscle mass; MVPA, moderate-to-vigorous physical activity.

### 3.2 Independent predictors of MHO and MUO

To identify the independent factors associated with MUO, variables showing significant associations in the univariate analysis (Table 2) were included in a multivariable logistic regression model. After completely adjusting for age, sex, geographical region, and pubertal development status (Table 3), FM, android FM, VAT, and FFM remained independently associated with MUO status under both CR and IR definitions. For each standard deviation (SD) increase in FM, the odds of MUO-CR and MUO-IR both increased by 21%. Each SD increase in android FM was associated with 41% higher odds of MUO-CR and 20% higher odds of MUO-IR. Similarly, a 1-SD increase in VAT increased the odds of MUO-CR by 27% and MUO-IR by 20%. For FFM, each SD increment elevated the odds of MUO-CR by 16% and MUO-IR by 17%.

**Table 3:** Independent risk factors for MUO under the two definitions.

	OR	95% CI	*p*-value
CR			
Fast food ≥1 time/week, %	1.36	1.00–1.85	0.053
Soft drinks ≥1 time/week, %	1.27	0.93–1.72	0.127
BMI z-score	1.63	1.38–1.95	<0.001
FM z-score	1.21	1.04–1.41	0.017
ANDROID_FM z-score	1.41	1.21–1.66	<0.001
TRUNK_FM z-score	1.39	1.18–1.63	<0.001
FM_arm z-score	1.14	0.99–1.33	0.080
VAT z-score	1.27	1.09–1.49	0.002
SAT z-score	1.26	1.05–1.51	0.013
FFM z-score	0.84	0.71–0.97	0.023
IR			
Sleep duration z-score, h/day	0.83	0.7–0.98	0.030
Father’s education university or higher, %	0.75	0.45–1.26	0.282
Mother’s education university or higher, %	0.70	0.41–1.21	0.202
BMI z-score	1.25	1.08–1.45	0.004
FM z-score	1.21	1.02–1.43	0.027
ANDROID_FM z-score	1.20	1.02–1.42	0.029
GYNOID_FM z-score	1.22	1.03–1.45	0.023
FM_limbs z-score	1.25	1.06–1.49	0.008
FM_arm z-score	1.16	1.00–1.37	0.058
VAT z-score	1.20	1.01–1.42	0.041
FFM z-score	0.83	0.70–0.98	0.031

ORs were adjusted for age, sex, puberty, region, and the significant variables from Table 2. MUO, metabolically unhealthy obesity; CR, cardiometabolic risk; IR, insulin resistance; BMI, indicates body mass index; WC, waist circumference; FM, fat mass; FFM, fat-free mass; VAT, visceral adipose tissue; SAT, subcutaneous adipose tissue.

Among behavioral factors, participants consuming fast food ≥1 time/week had 36% higher odds of MUO-CR compared to those with less frequent consumption. Longer sleep duration per SD was associated with 27% lower odds of MUO-IR.

### 3.3 Interactions between body composition and lifestyle behaviors of MHO and MUO

**Figure 2:**
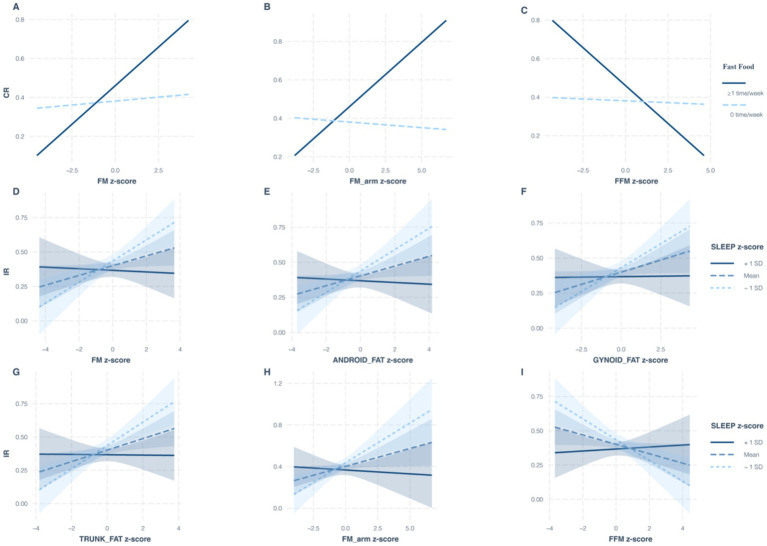
Influence of body composition and lifestyle interactions on the translation between MHO and MUO under different diagnostic criteria. Under the CR criteria, multiplicative interactions of fast food consumption with FM **(A)**, arm FM **(B)**, and FFM **(C)** on the MUO phenotype; under the IR criteria, multiplicative interactions of sleep duration with FM **(D)**, android FM **(E)**, gynoid FM **(F)**, trunk FM **(G)**, arm FM **(H)**, and FFM **(I)** on the MUO phenotype. MHO, metabolically healthy obesity; MUO, metabolically unhealthy obesity; FM, fat mass; FFM, fat-free mass; CR, cardiometabolic risk; IR, insulin resistance.

To investigate potential interactions between body composition and lifestyle behaviors on MHO and MUO phenotypes, we assessed the most significant lifestyle factors associated with each phenotype under different metabolic criteria. Specifically, fast food consumption was analyzed as a key dietary factor for MUO individuals meeting CR criteria. At the same time, sleep duration was evaluated as a critical behavioral factor for MUO individuals meeting IR criteria. As shown in Table 4, frequent fast food intake (≥1 time/week) was associated with an enhanced effect on MUO-CR in individuals with higher FM (odds ratio (OR) = 1.36, 95% confidence interval (CI): 1.01–1.82; *p* = 0.042) and arm FM (OR = 1.37, 95% CI: 1.02–1.83; *p* = 0.036). These findings suggest that FM and arm FM may potentiate the adverse effects of fast food intake on MUO-CR risk. In contrast, FFM (OR = 0.72, 95% CI 0.54–0.97; *p* = 0.032) appeared to attenuate these effects, indicating a protective role of lean body mass. For MUO-IR, sleep duration interacted multiplicatively with FM, android FM, gynoid FM, trunk FM, arm FM, and FFM (all *p* < 0.029) (Figure 2).

**Table 4:** Interaction of body composition and lifestyle factors in MHO and MUO under two different definitions.

	OR	95%CI	*p-*value
MUO-CR			
Fast food: FM z-score	1.36	1.01–1.82	0.042
Fast food: ANDROID_FM z-score	1.12	0.83–1.52	0.460
Fast food: TRUNK_FM z-score	1.34	0.99–1.82	0.058
Fast food: FM_arm z-score	1.37	1.02–1.83	0.036
Fast food: VAT z-score	1.09	0.81–1.46	0.559
Fast food: SAT z-score	1.23	0.92–1.65	0.164
Fast food: FFM z-score	0.72	0.54–0.97	0.032
MUO-IR			
Sleep duration z-score: FM z-score	0.81	0.71–0.93	0.003
Sleep duration z-score: ANDROID_FM z-score	0.87	0.75–0.99	0.046
Sleep duration z-score: GYNOID_FM z-score	0.88	0.78–1.00	0.046
Sleep duration z-score: TRUNK_FM z-score	0.83	0.72–0.95	0.010
Sleep duration z-score: FM_arm z-score	0.86	0.76–0.98	0.024
Sleep duration z-score: VAT z-score	0.95	0.83–1.08	0.431
Sleep duration z-score: FFM z-score	1.22	1.07–1.41	0.004

OR was adjusted for age, sex, puberty, and region. MHO, metabolically healthy obesity; MUO, metabolically unhealthy obesity; CR, cardiometabolic risk; IR, insulin resistance; FM, fat mass; FFM, fat-free mass; VAT, visceral adipose tissue; SAT, subcutaneous adipose tissue.

## 4 Discussion

Through this large-scale population study of Chinese children and adolescents, we demonstrate that over half of individuals with obesity in this population exhibit relatively low CR or IR despite an elevated BMI. This finding underscores the clinical relevance of identifying MHO phenotypes. Second, multivariable analyses revealed that body composition factors—including FM, gynoid FM, and VAT—as well as lifestyle behaviors (e.g., frequent fast food consumption and inadequate sleep duration) were significantly associated with MUO phenotypes. Finally, significant multiplicative interactions between body composition and lifestyle factors were associated with MUO, suggesting that their synergistic effects may substantially influence metabolic health in children and adolescents with obesity. Although previous research has independently established associations between either body composition or lifestyle factors and metabolic health, few studies have simultaneously examined the integrated role of both in a pediatric cohort.

Currently, no universally accepted definition exists for MUO in either pediatric or adult populations. Existing classifications predominantly rely on CR criteria—considered more clinically actionable (21)—or IR criteria, which reflect underlying pathophysiology (22). To enable direct comparison between phenotypes, we adopted both CR- and IR-based definitions. Literature reports show MHO prevalence ranging from 6 to 75% across studies (23, 24), largely due to heterogeneous metabolic health definitions and population characteristics. In our cohort of children, 56.4% met the MHO criteria by CR classification and 61.1% by IR classification. This substantial proportion of MHO cases highlights the necessity of incorporating metabolic heterogeneity into childhood obesity management frameworks, even in the absence of standardized diagnostic criteria.

The influence of behavioral factors on MHO and MUO phenotypes remains debated. Contrary to findings in adult populations (25), our study, consistent with other research in adolescents (26), found no significant differences in physical activity or sedentary time between the MHO and MUO groups. This discrepancy may be due to developmental differences; the detrimental metabolic effects of sedentary behavior might be more pronounced in adults, whereas in children, dietary habits may play a more critical role. Soft drink consumption is disproportionately high in developing countries (27). For instance, a Chinese cross-sectional study found that soft drinks contribute 10–15% of daily caloric intake among primary and secondary school students (28). The Global Burden of Disease Study links sugar-sweetened beverages (SSBs) to ectopic fat deposition, type 2 diabetes mellitus, cardiovascular disease, and related morbidities (29), with fast food consumption similarly associated with these outcomes (30). Our large-scale analysis demonstrates that SSB and fast food intake, combined with sleep duration, independently predict MUO status. Although adult studies suggest that sleep duration independently correlates with MUO (31), pediatric evidence remains scarce (32). These findings underscore the need to prioritize dietary interventions (limiting SSBs and fast food) and sleep hygiene in metabolic health management for children with obesity.

FM, android FM, VAT, and FFM were significant independent predictors under both the CR and IR criteria. This finding reinforces the critical role of body fat composition distribution, beyond simple BMI, in determining metabolic health status in youth. Notably, ASMM, arm ASMM, and leg ASMM, showed no significant association with metabolic status, suggesting that regional fat distribution and fat-free mass may play more dominant roles than peripheral muscle mass in early metabolic dysregulation. Our study further identified multiplicative interactions between fast food intake and FM (total and arm) as well as FFM, suggesting that the detrimental impact of a diet high in fast food on adiposity and potentially muscle mass may be amplified in individuals predisposed to MUO. Conversely, those with MHO might be more resilient to these effects in specific body compartments. This finding implies that dietary interventions targeting fast food reduction could be particularly important for managing body composition in youth at risk for MUO. The widespread interactions between sleep duration and nearly all FM regions (total, android, gynoid, trunk, and arm) as well as FFM are highly significant. Shorter sleep duration may promote unfavorable fat deposition or impair muscle metabolism, contributing to the MUO phenotype. These findings support the inclusion of sleep hygiene in interventions for children with obesity.

Therefore, clinical management of children and adolescents with MUO should integrate a comprehensive assessment of sleep patterns and dietary habits to optimize intervention efficacy. Importantly, MHO should not be perceived as a benign condition; our results emphasize that individuals with MHO require proactive strategies—including body fat regulation and lifestyle modification—to mitigate risks of metabolic sequelae such as cardiovascular disease. The results underscore significant associations between body composition, lifestyle factors, and MUO status and further reveal that these factors interact with each other. This finding deepens insights into obesity heterogeneity and provides a basis for precise interventions in childhood obesity. The strengths of our study, including a large sample size and the use of DXA for precise quantification of body composition, enhance the reliability of these findings.

However, this study has several limitations. First, although we adopted a rigorous multicenter study design and stringent inclusion criteria, all participating centers were located in urban areas. This may limit the representation of rural populations and introduce selection bias. Second, regarding measurement, despite implementing standardized data collection procedures (e.g., using unified measurement tools and trained surveyors) to maximize data quality, potential measurement bias may persist due to inter-surveyor variability and subtle equipment differences. Third, although we adjusted for key confounding factors (e.g., age, sex, pubertal stage, and region) in our analyses, other confounders from unmeasured variables (e.g., gut microbiota composition, psychological or behavioral factors) could influence the results. Finally, as a cross-sectional study, our design was unable to infer causal relationships or depict the dynamic trajectories of MHO and MUO states over time. Emerging evidence suggests that MHO is a transient state, with the potential to progress to metabolically healthy non-obese phenotypes (33, 34). Therefore, future cohort studies should focus on clarifying their long-term stability and health impacts.

## 5 Conclusion

This study highlights that fewer than 50% of children with obesity in China are affected by MUO. The results underscore significant associations between body composition, lifestyle factors, and MUO status. These insights provide a deeper understanding of the mechanisms behind metabolic dysfunction in obesity and could inform the development of targeted treatment strategies for pediatric populations with obesity.

## Data Availability

The raw data supporting the conclusions of this article will be made available by the authors, further inquiries can be directed to the corresponding authors.
